# Prehabilitation in Adult Cancer Patients Undergoing Chemotherapy or Radiotherapy: A Scoping Review

**DOI:** 10.3390/cancers18020286

**Published:** 2026-01-16

**Authors:** Dylan Kwan, Wesley Kwan, Anchal Badwal, Tuti Puol, Justin Zou Deng, Raymond Wang, Saad Ahmed, Alexandria Mansfield, Rouhi Fazelzad, Jennifer Jones

**Affiliations:** 1 Michael G. Degroote School of Medicine, McMaster University, Hamilton, ON L8S 4L8, Canada; dylan.kwan@medportal.ca; 2 Faculty of Health Sciences, Queen’s University, Kingston, ON K7L 3N6, Canada; wesley.kwan@queensu.ca (W.K.); tuti.puol@queensu.ca (T.P.); 22cb61@queensu.ca (R.W.); 3 Faculty of Kinesiology & Physical Education, University of Toronto, Toronto, ON M5S 2W6, Canada; anchal.badwal@mail.utoronto.ca; 4 School of Nursing, Queen’s University, Kingston, ON K7L 3N6, Canada; 22msc3@queensu.ca; 5 School of Public Health Sciences, University of Waterloo, Waterloo, ON N2L 3G1, Canada; s27ahmed@uwaterloo.ca; 6 Temerty School of Medicine, University of Toronto, Toronto, ON M5S 1A8, Canada; alexandriagrace.mansfield@uhn.ca; 7 Library and Information Services, University Health Network-Princess Margaret Cancer Centre, Toronto, ON M5G 2M9, Canada; rouhi.fazelzad@uhn.ca; 8 Cancer Rehabilitation and Survivorship Program, Princess Margaret Cancer Centre and Department of Psychiatry, University of Toronto, 200 Elizabeth Street, B-PMB-045, Toronto, ON M5G 2C4, Canada

**Keywords:** prehabilitation, cancer, chemotherapy, radiotherapy, non-surgical treatment

## Abstract

**Purpose**: The effectiveness and feasibility of cancer prehabilitation have been well-validated in surgical settings, but its role in non-surgical treatments, such as chemotherapy and radiotherapy (RT), remains unclear. This scoping review aims to systematically explore the existing literature on prehabilitation programs for non-surgical cancer treatments. **Methods**: Following the scoping review methodology developed by the Joanna Briggs Institute, seven databases were systematically searched from their inception to October 2024 for peer-reviewed studies involving prehabilitation prior to non-surgical treatment. Data were extracted and reported adhering to PRISMA-ScR guidelines, using a convergent synthesis design to present qualitative and quantitative evidence. No formal risk-of-bias or quality appraisal was conducted. **Results**: Of 22,122 studies, 39 met the inclusion criteria, yielding a combined sample of 6073 patients and thirty-four unique interventions. Sample sizes ranged from 9 to 1992, with randomized control trials being the most common (16). Head and neck cancer was the most frequently studied, followed by breast, esophageal/gastric, and lung cancer. Of the included interventions, 23 were unimodal and 16 were multimodal. Exercise was the most common component (30), with nutrition (13), psychosocial (10), and educational (8) components also present. Most efficacy studies (84%) reported improved outcomes and nearly all (93%) feasibility studies found prehabilitation acceptable and implementable. **Conclusions**: This review highlights a growing body of literature examining prehabilitation prior to chemotherapy or RT in adult cancer patients, with studies suggesting potential benefits and feasibility. However, long-term trials, especially in diverse cancers and older populations, remain scarce. Our results provide insight into future implementation, evaluation of outcomes, and directions for future prehabilitation research.

## Simple Summary

Individuals undergoing cancer treatment often experience side effects like fatigue, muscle loss, and mood changes that can reduce their ability to carry out daily activities. In surgical settings, giving patients a prehabilitation program involving exercise, nutrition, and psychological support prior to treatment helps preserve their strength and quality of life, yet its use for non-surgical treatments remains largely unexamined. Our review therefore examines research on prehabilitation before non-surgical treatments, such as chemotherapy and radiotherapy, to see what kinds of programs exist, how feasible they are, and which patient groups benefit. By mapping the evidence and identifying gaps, we aim to guide clinicians and researchers toward designing better pre-treatment support programs and highlight the need for longer-term trials in diverse and older populations.

## 1. Introduction

Advances in cancer treatments have enabled more patients to live with and beyond cancer [1]; however, these therapies often cause adverse effects and functional impairments that reduce quality of life (QoL) and psychosocial well-being. Radiotherapy (RT) and chemotherapy can cause long-term mental and physical health consequences [2], such as mucositis and weight loss in head and neck chemoradiation, diarrhea and dehydration in gastrointestinal chemoradiation, and fatigue and impaired vision with brain treatments [3,4]. Chemotherapy-induced musculoskeletal degradation increases the risk of falls and fractures, leading to long-term immobility [5,6]. Other side effects—including fatigue, anxiety, depression, and apathy—diminish survivors’ functional status and QoL [2,7].

Functional status reflects a person’s ability to perform activities of daily living (ADLs) and meet basic needs. Functional decline often accelerates following cancer diagnosis and treatment, largely due to treatment-associated muscle and bone loss and deconditioning [8,9]. This decline not only reduces QoL but also increases comorbidity burden, mental health concerns, caregiver dependency, and institutionalization [10,11,12]. Therefore, addressing cancer treatment-related adverse effects is crucial to improving the well-being of cancer survivors.

Prehabilitation (“prehab”) has emerged so far as an effective intervention for improving post-treatment functional outcomes in cancer patients [13]. It takes a proactive approach, implemented between diagnosis and the start of acute treatment [14]. Prehab begins with establishing a baseline functional level, after which clinicians provide physical and psychological interventions to limit future impairments [14]. Current approaches discourage a “one-size-fits-all” model, emphasizing individualized, goal-directed care [15]. Accordingly, many programs adopt a multimodal design. The most common components are exercise and nutrition, sometimes combined with occupational therapy or psychological support [14,16]. Ultimately, prehab aims to preserve functional status, enhance physical and mental well-being, and increase patients’ capacity to undergo oncology treatments [14,17,18].

In surgical settings, cancer prehab programs have been largely well-validated, with studies demonstrating the feasibility and efficacy of both stand-alone exercise programs and multimodal interventions incorporating exercise, nutrition, and psychological support [13,19,20]. Such interventions reduce hospital stays and improve post-operative recovery. In contrast, evidence for prehab in non-surgical contexts such as RT and chemotherapy remains limited, with no comprehensive review of these modalities [14,21].

This scarcity of prehabilitation research is particularly concerning because certain populations may benefit the most. Older adults are more likely to receive non-surgical therapies such as chemotherapy, RT, or immunotherapy due to lower baseline physical status [14,22]. Designing a prehab program for this group requires tailored considerations, including prioritizing safety, feasibility, and offering home-based exercise options [23]. While prehab programs have historically emphasized aerobic exercises, there is growing recognition of the high prevalence of sarcopenia and muscle loss in older cancer patients [14,23,24]. Consequently, evidence supports prioritizing resistance training and protein supplementation to improve muscle function in this population. Yet the lack of research makes it difficult to determine the full extent of these benefits, whether other populations may also benefit, and how best to implement such programs.

The goals for prehabilitation often differ between surgical and non-surgical contexts. For example, in chemotherapy or RT, prehab primarily aims to improve treatment tolerance and adherence, enhance long-term survival, and maintain QoL by reducing side effects and toxicities [14]. The longer treatment periods associated with these modalities also create opportunities to integrate prehabilitation before treatment with rehabilitation during or after therapy in non-surgical settings [25]. Interventions for patients undergoing RT or chemotherapy are recommended to continue as long as possible, with ongoing risk assessment to ensure safety. Timing is another key consideration: patients who travel frequently for radiation sessions or chemotherapy may be better suited for at-home rather than community-based programs [14,26]. These differences highlight the need for a comprehensive review of the current literature to identify best practices and research gaps in non-surgical cancer prehabilitation.

Scoping reviews are particularly valuable when a body of literature has yet to be comprehensively reviewed [27], as is the case for the heterogeneous and conceptually fragmented literature in this subfield. This review maps evidence on prehabilitation programs for non-surgical cancer treatments, with a primary focus on chemotherapy and RT. The goal is to inform clinical practice while appropriately contextualizing reported outcomes within the exploratory nature of the current evidence base, since integrating prehabilitation before chemotherapy or RT may improve therapeutic tolerance and treatment-related outcomes.

## 2. Materials and Methods

Our methodology was developed in accordance with the framework proposed by Arksey and O’Malley [28], later updated by the Joanna Briggs Institute (JBI) [27]. The reporting of our scoping review was guided by the Preferred Reporting Items for Systematic Reviews and Meta-Analysis extension for scoping reviews (PRISMA-ScR) [29,30]. Our review was registered on OSF: https://doi.org/10.17605/OSF.IO/7DKRS.

**Stage 1: Identifying Research Questions**

The scoping review answers the following questions:

- What current forms of prehabilitation are used for non-surgical cancer treatments?
- What feasibility, implementation strategies, and outcomes have been found for these prehabilitation interventions?
- Which populations benefit the most from non-surgical cancer prehabilitation?
- What gaps exist in the current literature, and where is future research needed?

**Stage 2: Identifying Relevant Studies**

### 2.1. Eligibility Criteria

Clear inclusion and exclusion criteria (Table 1) were developed, which follow the Population, Concept, and Context categories for scoping reviews. The review focuses on peer-reviewed articles published in English, with no restrictions based on the date of publication. For the purposes of this study, prehabilitation was defined as any intervention in which at least one component or a subset of participants initiated the intervention prior to the start of non-surgical cancer treatment, with no restrictions based on the intervention’s end date. While this broad operational definition may overlap with early rehabilitation, particularly for interventions extending into cancer treatment, this inclusivity captures the full scope of prehabilitation practices reported in the literature.

### 2.2. Search Strategy

In collaboration with the information specialist, an extensive literature search was conducted in Medline ALL (Medline and Medline Epub Ahead of Print and In-Process & Other Non-Indexed Citations), Embase Classic +Embase, Emcare, Cochrane Central Register of Controlled Trials, PsycInfo all from the OvidSP platform, and Scopus from Elsevier, from the database’s inception to October 2024. Where available, each search strategy included a combination of controlled vocabulary terms and text words, adapting the database-specific search syntax. The search was restricted to human studies, adults, and English publications, excluding books, conferences, dissertations, reviews, and preprints. The Medline search strategy can be found in the Supplementary Information. This review focuses exclusively on identifying and analyzing primary/original research published in peer-reviewed journals due to the heterogeneity of study designs and the need for consistent methodological standards in this emerging field; therefore, gray literature was not included to enhance cross-study comparability. The reference lists of relevant review papers were hand-searched for relevant articles.

**Stage 3: Study Selection**

Results from the literature search were imported into Covidence, a web-based collaboration software platform for duplicate removal and screening [31]. The selection of studies was conducted in a two-stage screening process. First, two independent reviewers screened the titles and abstracts according to the predefined inclusion and exclusion criteria. Any conflicts between the two reviewers during this phase were resolved by a third team member. The second stage involved a full-text review. Similar to the first stage, two independent reviewers screened all articles to determine whether they met the inclusion criteria. Conflicts arising during this phase were discussed and resolved by a third team member. A PRISMA 2020 flow diagram was used to illustrate the review process for screening and the reason for exclusions [32].

**Stage 4: Data Extraction**

A pre-defined data extraction form based on the key principles from JBI’s template for data extraction was used [27]. The extraction process was carried out independently by two reviewers, and any conflicts were resolved by a third team member. Information extracted from each study includes the domains highlighted in Table 2. Recommendations regarding what populations may benefit the most from these interventions, as well as qualitative information regarding participants’ views or opinions on the prehab interventions, were noted. Given that the goals of this scoping review include identification and summarization of research gaps and opportunities, risk-of-bias and quality of evidence assessments of each study were not conducted. To clarify intervention timing, corresponding authors of studies screened in the full-text review were contacted to determine whether an approximate prehabilitation start date relative to treatment initiation could be ascertained.

**Stage 5: Collating, Summarizing, and Reporting Results**

All extracted data were summarized to provide a comprehensive overview of the collected literature. Descriptive analyses were performed, including frequencies and central measures of tendency that report on the number and proportion of studies under each population category, prehabilitation intervention used, key study characteristics, and outcome results.

The reporting and interpreting of all collated data were guided by the main objectives of this scoping review, namely (a) determining the implementation, feasibility, and efficacy of current non-surgical cancer prehab programs, (b) identifying current prehab programs that exist, (c) determining which populations benefit the most from these programs, and (d) identifying gaps in the current literature. A parallel-results convergent synthesis design was used to report qualitative and quantitative evidence [33]. In this approach, quantitative and qualitative data were analyzed separately but interpreted together during the discussion. When analyzing quantitative studies/results, our primary focus was on presenting reported benefits or drawbacks of specific prehabilitation programs on health-related outcomes. In qualitative analysis, we aimed to identify major themes across the included studies concerning the feasibility of non-surgical prehab programs, patient preferences and opinions on these interventions, and the barriers and enablers to their implementation.

## 3. Results

### 3.1. Search

**Figure 1:**
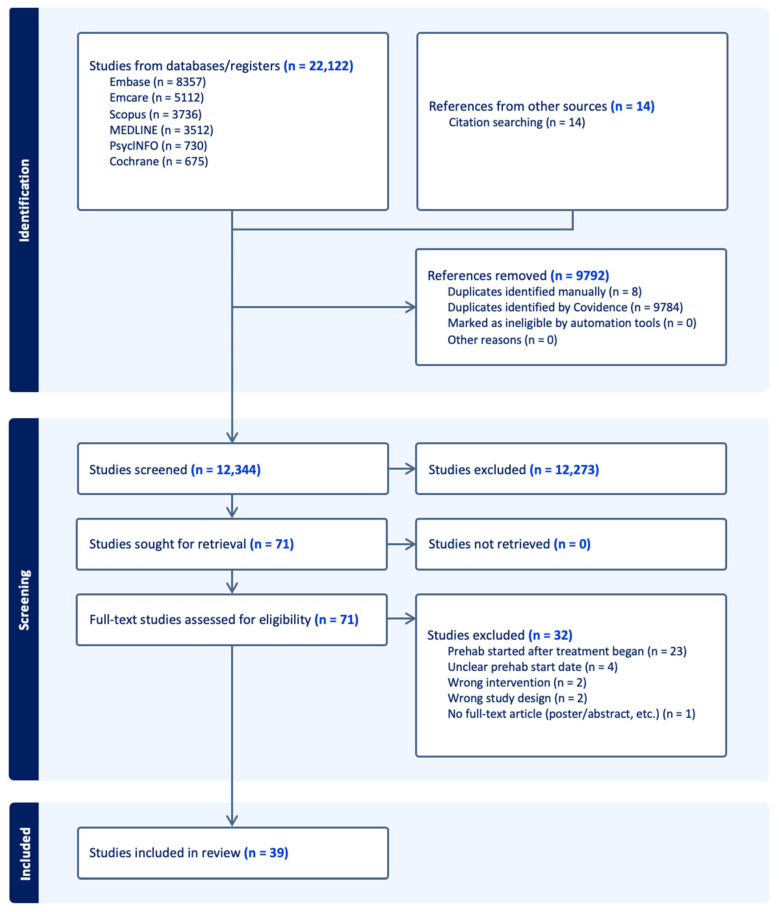
Prisma-ScR flow chart of included studies.

The search yielded 22,122 studies, resulting in 12,344 studies after duplicates were excluded. Following title and abstract screening according to the inclusion criteria, seventy-one studies remained. During full-text review, 32 studies were excluded, leaving a total of 39 included studies (Figure 1).

### 3.2. Characteristics of Included Studies

There was a combined sample of 6073 patients across various non-surgical oncological settings, with sample sizes ranging from 9 to 1992 participants. The included studies were diverse in study design as follows: 16 (41%) were randomized control trials (RCTs) [34,35,36,37,38,39,40,41,42,43,44,45,46,47,48,49], 13 (33%) were prospective cohort or single-arm studies [50,51,52,53,54,55,56,57,58,59,60,61,62], 6 (15%) were retrospective studies [63,64,65,66,67,68], 2 (5%) were economic intervention evaluations [69,70], 1 (3%) was a non-randomized matched-pair study [71], and 1 (3%) was an implementation report [72]. Thirty studies had comparison groups, while nine studies did not. Of the studies with comparison groups, 29 were standard care and 1 compared different age groups [68].

Ten studies involved patients receiving neoadjuvant chemotherapy [34,35,40,41,55,56,62,65,66,72], while six studies involved adjuvant chemotherapy [37,48,49,54,61,72]. Eleven studies involved patients receiving adjuvant RT [37,50,51,52,53,54,60,63,67,71,72], ten studies involved definitive RT [36,38,43,50,51,53,59,60,63,71], and two studies involved unspecified RT [39,57]. Seven studies involved neoadjuvant combined chemoradiotherapy (CTRT) [40,41,44,45,56,65,66], six studies involved adjuvant CTRT [51,53,63,64,67,68], and fifteen studies involved definitive CTRT [36,38,42,43,46,47,50,51,53,60,63,64,67,69,70]. One study involved an unspecified radical anticancer treatment [58]. Some studies were included in multiple treatment categories because their participants received different cancer treatments (see Table 3).

**Figure 2:**
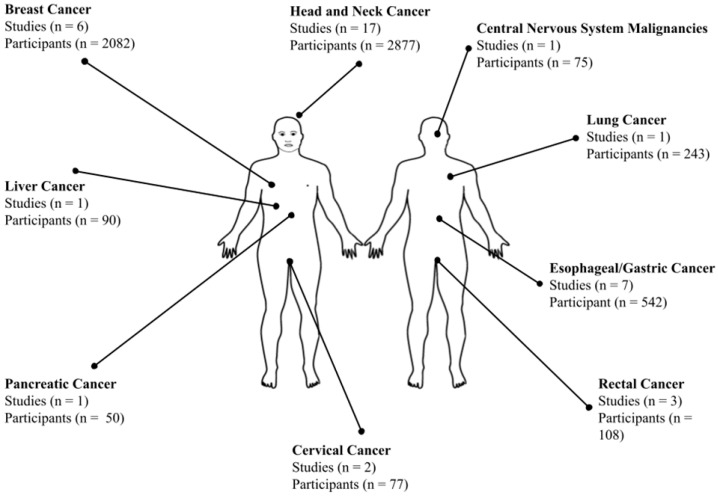
Organizational diagram of study and participant numbers for each cancer type based on anatomical location, comprising the included studies of our review.

Depicted in Figure 2, the most commonly studied population were patients with head and neck cancer (HNC) (17) [36,38,42,43,46,47,50,51,52,53,60,63,64,67,69,70,71], followed by esophageal/gastric (7) cancer [34,40,41,55,62,65,66], and breast (6) cancer [35,37,48,49,54,72]. Fewer studies addressed prehab in rectal (3) [44,45,61], lung (1) [58], pancreatic (1) [56], liver (1) [39], cervical (2) [57,59], or central nervous system malignancies (1) [68]. All studies involved adult populations, with the median ages ranging from 44 to 70 years. Only one study reported on age-specific outcomes [68].

Most studies (32) assessed efficacy outcomes, of which 27 (84%) studies reported beneficial effects or improvements, such as improved functional capacity, symptom control, or treatment tolerance [34,35,38,39,40,42,45,46,47,48,49,51,52,54,55,56,57,58,62,63,64,65,66,67,68,69,71]. In contrast, five (16%) studies did not observe significant improvements or were inconclusive [37,41,43,50,59]. In addition, feasibility outcomes were examined by 15 studies, with 14 (93%) generally reporting high feasibility [35,36,44,46,47,48,52,53,59,60,61,63,64,72], while only 1 (7%) study raised notable feasibility concerns, reporting high dropout rates and moderate compliance [43]. Two studies evaluated cost-effectiveness outcomes for the same intervention, both of which found the prehabilitation approach to be cost-effective [69,70]. There was an overlap among studies, with some studies assessing both efficacy and feasibility or cost-effectiveness. Reported outcomes (e.g., efficacy, cost-effectiveness, etc.) were drawn from a highly heterogeneous body of included study designs (e.g., small RCTs, retrospective cohort studies, etc.) with inherent confounding variables and are presented as reported findings without weighting by methodological quality or effect size.

By country, 8 studies were conducted in the United Kingdom [36,44,45,55,58,62,65,66], 6 in the United States [38,42,56,57,60,63], 6 in the Netherlands [46,47,53,61,69,70], 3 in Italy [51,54,72], 2 each in Australia [52,64], Canada [35,67], China [39,49], and Ireland [40,41], and 1 each in India [50], Lithuania [34], Germany [71], Sweden [37], Denmark [43], Japan [68], Chile [59], and Taiwan [48].

### 3.3. Intervention Characteristics

A majority of studies implemented only a single prehab intervention component, with 23 (59%) unimodal intervention studies and 16 (41%) multimodal intervention studies. Of the 39 studies, there were 34 distinct or unique interventions. Multiple studies analyzed outcomes for the same intervention program, resulting in 3 interventions encompassing 8 studies as follows: Loughney et al. [40] and Loughney et al. [41] share the same intervention program; Moug et al. [44] and Moug et al. [45] share the same intervention; and Retel et al. [69], Retel et al. [70], van der Molen et al. [46], and van der Molen et al. [47] share the same intervention. Thirty (88%) interventions incorporated an exercise component [34,35,36,38,39,40,41,42,43,44,45,46,47,48,49,50,51,52,53,55,56,57,58,59,60,61,62,63,65,66,68,69,70,71,72], thirteen (38%) interventions included nutrition or dietary support components [34,35,39,54,56,58,61,64,65,66,67,71,72], ten (29%) interventions provided psychosocial support or psychological intervention [34,35,36,39,49,58,65,67,68,72], and eight (24%) interventions had an educational component [36,39,49,53,56,60,67,72]. Given the existence of multimodal interventions, some interventions span multiple component categories. For the purposes of this review, exercise-only interventions were classified as unimodal regardless of whether they involved single or combined exercises, and analyses of more detailed characteristics such as exercise intensity, frequency, and adherence were considered beyond the scope of this synthesis. For a comprehensive breakdown of each study’s baseline characteristics, intervention details, and outcomes, see Table 3 and Table 4.

## 4. Discussion

This scoping review synthesizes 13 years of research across sixteen countries, thirty-nine studies, and thirty-four unique non-surgical cancer prehabilitation interventions, evaluating implementation, feasibility, and outcomes in routine care. The studies varied in design, participant demographics, cancer and treatment type, and outcome measures. Key themes, such as intervention domains, cancer contexts, patterns in outcomes, and age considerations, can help contextualize current practices and inform future exploration of prehabilitation in chemotherapy and RT. Our findings describe implementation strategies, identify barriers, and highlight reported outcomes for patient evaluations.

### 4.1. Exercise Interventions

Exercise was the most common intervention, appearing in 30 out of 34 (88%) programs, with 16 being unimodal. Modalities varied by cancer type and treatment context, but general exercise programs were the most common. Nearly all exercise-based interventions included low-to-moderate aerobic activity, with walking programs being the most frequent, appearing in seven interventions. At least fifteen interventions also incorporated strength or resistance training. For example, Natsume et al. [68] employed walking and treadmill exercises, whereas Liu et al. [39] incorporated Baduanjin, a Chinese mind–body practice. Despite heterogeneous delivery and cultural tailoring, aerobic exercise remains the most common intervention. Future research should focus on resistance training, particularly for frail or sarcopenic populations that would benefit from improved muscle [14]. High-intensity interval training has also proven safe pre-surgery and may provide efficient aerobic gains [73], warranting further exploration in more physically fit cohorts.

Targeted swallowing exercises were present in all HNC interventions (12/30). Most, including Kotz et al. [38], used standardized exercises like the Effortful Swallow, tongue base retraction, Super-Supraglottic Swallow, and the Mendelssohn maneuver, introduced prophylactically to preserve swallowing and prevent dysphagia. Given that over 90% of HNC patients receiving RT experience treatment-related side effects such as oral discomfort, mastication difficulties, or speech and swallowing impairments [74], prehabilitation has the potential to mitigate these issues. At least six interventions incorporated technological adjuncts, notably TheraBite [46,47,69,70], a handheld unit to stretch the jaw for trismus, dysfunction, and hypomobility; Restorabite [52], a novel jaw stretcher; and Vibrent [60], a mobile application designed to enhance adherence to swallowing exercises. These tools were associated with favorable outcomes beyond traditional swallowing exercises, helping overcome common barriers like pain, fatigue, or lack of motivation.

Only two studies evaluated targeted pelvic exercise interventions. O’Loughlin et al. [57] assessed hip extension and external rotation exercises in gynecologic cancer patients undergoing pelvic RT. The intervention group demonstrated significantly lower sacral-slope variability (0.91° vs. 2.27°; *p* = 0.0001), indicating improved pelvic stability. Sacomori et al. [59] implemented a twice-daily home-based program for cervical cancer patients, including slow and fast pelvic floor contractions and the “knack,” a pre-emptive pelvic floor contraction before activities that increase intra-abdominal pressure. The intervention was reportedly feasible and showed potential benefits for pelvic floor strength and activation, but no significant post-treatment gains were observed. Despite pelvic RT’s known impact on musculoskeletal and functional outcomes, there is a notable literature gap regarding pelvic interventions [75].

### 4.2. Nutrition Intervention

Nutrition-focused prehabilitation appeared in thirteen (38%) interventions, targeting cancers with high treatment-related malnutrition risk, including gastric (3), head and neck (3), and breast (3), liver (1), pancreatic (1), lung (1), and colorectal cancers (1). A systematic review found cachexia rates were the highest among liver (50%), pancreas (45.6%), and HNC (42.3%) patients [76]. The underrepresentation of liver and pancreatic cancers suggests that future studies should prioritize nutrition-based prehabilitation for these high-risk groups.

Most nutrition programs targeted treatment-related side effects on nutritional status. For example, Büntzel et al. [71] combined IV parenteral nutrition, oral nutrition, and swallowing exercises in HNC patients, reducing RT interruptions, toxicities, and improving functional outcomes. In liver cancer, Liu et al. [39] found that a multimodal program with nutritional assessment and microecological supplementation (using microorganisms to balance the human microbiota) improved nutritional status, immune function, RT resistance, and perioperative QoL. Among esophageal/gastric cancer patients receiving NAC, combined exercise and nutritional therapy improved chemotherapy completion rates (93.6% vs. 77.7%) [65]. However, the multimodal designs limit the ability to isolate the specific contribution of nutrition.

Two studies employed unimodal nutrition-focused interventions. Blake et al. [64] provided dietetic counseling and enteral nutrition via gastrostomy for HNC patients undergoing NAC, reporting an insignificant reduction in weight loss and nutritional decline. Interestingly, targeted nutritional interventions may offer benefits beyond traditional markers like weight or muscle mass. A cohort study [54] of breast cancer patients reported that a 6-week antioxidant regimen reduced radiation dermatitis compared to standard topical care. Together, these findings highlight the potential of nutritional prehabilitation to improve outcomes across cancer types and treatments. However, larger studies are needed to clarify efficacy.

### 4.3. Psychosocial Support and Educational Interventions

Psychosocial support and patient education were key components in a quarter of prehabilitation programs, typically within multimodal interventions alongside exercise or nutrition. Pre-treatment education was the most common, delivered via coaching, printed materials, or digital platforms to improve treatment understanding, promote self-management, and enhance adherence. A feasibility trial [36] in HNC combined a video education package with behavior change strategies (e.g., goal setting, self-monitoring), resulting in high patient engagement and adherence to swallowing exercises. Similarly, the Vibrent mobile application supported swallowing exercise adherence with video demonstrations, daily reminders, pain and weight logging, a messaging system, and adaptive exercise dosing based on self-reported pain [60].

Targeted psychosocial interventions, such as smoking cessation, occupational therapy, stress management, and meditation, were also represented in ten interventions. For instance, Xu et al. [49] conducted an RCT on a large Mindfulness-Based Cancer Recovery program for breast cancer patients undergoing chemotherapy. The program included mindfulness, breathing exercises, guided walking, acceptance strategies, and symptom management, reducing anxiety, depression, PTSD, and fatigue. Depression affects 27% of cancer patients globally [77], highlighting the importance of addressing psychological well-being. Psychoeducation interventions also have the potential to improve clinical outcomes. Malik et al. [67] reported that a psychoeducational prehab class on swallowing dysfunction, nutrition, and hydration in HNC patients was linked to higher survival rates and fewer RT complications. Implementation data further support patient interest in psychosocial and educational support. Rossi et al. [72] described an integrative oncology prehabilitation clinic in Italy, where 83% of 1500 breast cancer patients voluntarily received lifestyle counseling, 85% participated in psycho-oncological consultations before NAC, and many engaged in complementary therapies. This suggests a strong interest in supportive interventions and aligns with the benefits and feasibility of psychosocial/education prehabilitation strategies.

### 4.4. Cancer Types Represented

Cancer types studied were imbalanced. HNC was the focus of 17 out of 39 studies (44%), which is disproportionately high compared to its 4.5% share of all cancer diagnoses and deaths [78]. This emphasis is justified by CTRT functional impairments [79] and high remission rates (80–90%) [80] in HNC, making it a logical target. Other cancers were less frequently studied, including the following: upper gastrointestinal cancers (7 studies), breast cancer (6), and fewer than 3 studies each for rectal, cervical, lung, liver, pancreatic, and brain cancers. Hematologic cancers were not represented. This gap may stem from practical constraints, as the median time from diagnosis to treatment initiation in hematologic cancers is as short as 5 days [81], limiting prehabilitation opportunities. In contrast, for HNC and other solid tumors, evidence suggests that delays between diagnosis and treatment may improve overall survival [82], allowing more time for prehabilitation. Future research should expand prehabilitation to more cancer types, particularly those with high symptom burden or treatment-related functional decline.

### 4.5. Treatment Types

Most prehabilitation studies focused on neoadjuvant or adjuvant chemotherapy and/or RT, with fewer studies on patients not undergoing surgery. Most interventions were delivered in the perioperative setting, often overlapping with other phases of care. Many studies included heterogeneous patient groups undergoing mixed treatment regimens (e.g., some with surgery, others definitive CTRT), making it challenging to isolate prehabilitation effects within specific treatment pathways.

Only one study, Liu et al. [39], implemented a time-limited prehabilitation program (10–15 days) prior to non-surgical treatment initiation (RT), aligning with the definition of “true” prehabilitation [83]. In all other studies, the intervention extended into or beyond the active treatment period, complicating efforts to attribute outcomes solely to the pre-treatment phase. This is particularly relevant in non-surgical oncology, where the short interval between diagnosis and treatment limits the feasibility of structured prehabilitation. Interventions starting before treatment and continuing during it may offer sustained clinical benefit, but vulnerable populations may be ineligible for chemotherapy at diagnosis [84]. Research is needed to determine whether prehabilitation before treatment could enable patients to become eligible, rather than merely improving post-treatment outcomes.

These findings also raise questions about prehabilitation’s definition and implementation in non-surgical oncology. In our review, we defined prehabilitation as interventions starting before active treatment, even if they extended into it. Other reviews included prehabilitation starting after RT [16,85]. Definitional variability led to the exclusion of twenty-three studies at the full-text screening stage, as they delivered interventions exclusively during treatment. As a result, evidence is limited on whether strictly pre-treatment programs offer distinct advantages. Further research is needed to distinguish the effects of true prehabilitation from broader supportive care models that extend into treatment. Additionally, studies focused on patients receiving definitive chemotherapy or RT without surgery are needed to enhance generalizability to populations with unresectable or advanced disease.

### 4.6. Study Outcomes

Study outcomes differed significantly based on study design, main questions of interest, and the cancer site studied. Overall, studies reported positive results, with only 6 of 39 studies failing to achieve significant improvements in their primary outcomes.

Prehabilitation was frequently associated with improvements in QoL and physical functioning, which typically decline during treatment. Of 10 studies measuring health-related QoL (HRQoL), 8 reported improvements, whereas 2 showed no difference compared to controls [37,43]. Ngo-Huang et al. [56] demonstrated significant gains in physical function and HRQoL with a home-based prehabilitation regimen in pancreatic cancer patients, despite many patients being older or frail. Of the two studies reporting no QoL improvement, Mortenson et al. [43] attributed the lack of effect to poor adherence and high dropout rates due to fatigue in HNC patients. In contrast, Heiman et al. [37] suggested the lack of improvement was due to already high baseline QoL in breast cancer patients, noting most returned to baseline QoL by 12 months post-surgery. Objective functional capacity and targeted outcomes were also positively impacted. Prehabilitation helped preserve or improve exercise capacity and body composition during therapy [34,35,39,40,48,55,66,68], while targeted swallowing exercises improved function and reduced reliance on feeding tubes [38,42,50,51,52,63,69]. However, not all studies showed significant improvements across all metrics. For instance, Loughney et al. [40] observed improved physical fitness (6MWT) due to an exercise program in esophageal cancer patients, but no impact on body composition or sedentary behaviors.

Although fewer studies focused on treatment tolerance and completion rates, those included reported a positive impact of prehabilitation on therapy adherence and treatment-related complications. In gastrointestinal cancers, a multimodal exercise and nutrition prehabilitation program improved chemotherapy completion rates and reduced dose reductions or delays [65]. Büntzel et al. [71] similarly found that a multimodal exercise and nutrition program in HNC patients reduced RT interruptions and toxicities. Targeted nutritional interventions, such as antioxidant supplementation, also reduced specific RT-adverse events like radiation dermatitis [54]. Interestingly, preliminary evidence suggests that prehabilitation may improve selected oncologic outcomes. Zylstra et al. [62] found that a unimodal exercise program before and during NAC increased tumor regression and nodal downstaging, possibly due to improved muscle mass and immune markers. A few studies reported improved survival outcomes, such as recurrence-free survival in HNC [67] and short-term survival in lung cancer [58], but results were inconsistent and limited by short follow-up. Overall, these findings suggest non-surgical prehabilitation may improve treatment tolerance and clinical endpoints, but larger trials with longer follow-up are needed to confirm these effects.

Feasibility was frequently assessed, particularly in pilot and single-arm trials, with 14 of 15 studies reporting prehabilitation as feasible. High patient engagement, adherence, and satisfaction were consistently observed, especially when supported by technology and educational tools. Technology-assisted programs, like mobile applications and digital platforms, improved accessibility, self-monitoring, and patient–provider communication. For instance, exit interviews showed the Vibrent application fostered accountability and improved communication, though feedback suggested enhancing customization and notification features [60]. A large-scale study by Rossi et al. [72] supported embedding multimodal prehabilitation into routine care, with high patient participation. Cost-effectiveness analyses of swallowing therapies in HNC demonstrated significant healthcare savings due to improved outcomes and fewer complications [46,47,69,70]. Patient satisfaction was commonly reported with positive experiences across sites [44,61]. For example, Brahmbhatt et al. [35] found high acceptability and emotional benefit in their multimodal prehabilitation program for breast cancer patients, which included home-based exercise, nutritional support, stress management, and smoking cessation. Cnossen et al. [53] noted that social support and perceived physical improvement facilitated adherence to swallowing exercises, while fatigue, poor baseline health, and low motivation impeded participation. Mortenson et al. [43] raised feasibility concerns about moderate adherence and high dropout rates in HNC patients undergoing RT. Collectively, non-surgical prehabilitation was reported to be generally feasible and well-accepted, though challenges remain in populations with significant treatment burden or frailty.

### 4.7. Age-Specific Considerations

Older adults are more likely to undergo non-surgical cancer treatment due to reduced physical status and treatment tolerance, making age an important consideration in prehabilitation planning. Although adults aged ≥85 years account for only 8% of all new cancer diagnoses, they represent nearly 17% of cancer-related deaths [86]. In our review, participant ages ranged from 44 to 70 years, with most studies (nineteen) including those under 60, and none with a median/mean age over 70. This limits generalizability to older populations, especially given the median age at diagnosis for colorectal cancer is over 70 [87], and 50% of HNC patients are above 70 years old [88]. Of the thirty-nine studies, only Natsume et al. [68] stratified by age, finding that prehab during adjuvant CTRT improved functional outcomes and activities of daily living, regardless of age. While adverse events were more common in the older group, no significant difference was seen in median overall survival (18.7 months older vs. 18.3 months younger age group; *p* = 0.87). Although Natsume et al. [68] reported a mean age of 72.5 years, it remains unclear if these results apply to patients ≥75 years, typically seen in a geriatric setting.

### 4.8. Limitations

Our scoping review had several limitations that suggest directions for future research. Most notably, due to the limited number of studies implementing prehabilitation exclusively before non-surgical treatment initiation, we adopted an inclusive definition of prehabilitation: any intervention with at least some component or participants starting prior to treatment. However, even within individual studies, variability in intervention timing further complicated interpretation, potentially overlapping with early rehabilitation. Additionally, certain cancer types (e.g., hematologic, brain, and pancreatic) and older populations were underrepresented. Few studies conducted age-stratified analyses, limiting age-specific conclusions. While our broad inclusion criteria captured a wide range of study designs, it also resulted in substantial heterogeneity across cancer types, patient populations, and treatment regimens, requiring cautious interpretation of aggregated findings.

As a scoping review, our goal was to map the existing literature rather than quantify prehabilitation effectiveness. Accordingly, we did not conduct a formal risk-of-bias or quality appraisal of individual studies, so we cannot grade the quality or certainty of evidence. While consistent with scoping review methodology, some findings may be drawn from lower-level or uncontrolled studies and should be interpreted with qualification. Our search was limited to English publications, potentially excluding relevant studies in other languages. Despite an extensive search, unpublished data may have been missed, and gray literature was excluded, introducing a risk of publication bias. Finally, while our review focused on non-surgical prehabilitation, many studies included peri-operative care components, limiting their relevance to purely non-surgical contexts. Our summary omits details or includes generalizations necessary for conciseness. We encourage readers to consult the full source articles.

## 5. Conclusions

This scoping review highlights the growing interest in prehabilitation for patients undergoing non-surgical cancer treatments such as chemotherapy or RT, with most studies focusing on neoadjuvant or adjuvant settings. Interventions were generally described as feasible, well-accepted, and associated with improvements in both patient-reported and objective outcomes. HNC was the most commonly studied cancer type, and low-to-moderate aerobic activity emerged as the primary intervention component, while other cancer types and modalities remain underexplored. Technological adjuncts (e.g., mobile applications) were reported as potential facilitators in enhancing participant engagement and scalability. However, results should be interpreted with caution, as the reviewed studies had heterogeneous designs and did not undergo a formal risk-of-bias or quality assessment. Nevertheless, these findings provide a broad scope of the reported evidence surrounding prehabilitation’s efficacy and feasibility and can be used to generate future hypotheses. Ultimately, larger, well-controlled trials with longitudinal follow-up are needed to better evaluate efficacy and long-term outcomes, especially in older or frailer populations. Finally, a lack of consensus on prehabilitation definitions and implementation in non-surgical oncology highlights the need for clearer conceptual frameworks.

## Figures and Tables

**Table 1:** Study inclusion and exclusion criteria.

	Inclusion Criteria	Exclusion Criteria
Population	Adults aged 18 or over	Individuals under the age of 18
	Individuals diagnosed with any form of cancer	Individuals without cancer
	Patients undergoing non-surgical cancer treatments, such as chemotherapy, RT, chemoradiation, immunotherapy, or hormonal therapy	Patients exclusively undergoing surgical cancer treatments without any neoadjuvant/adjuvant therapies
Concept or Focus	Any type of prehab program initiated prior to the onset of non-surgical cancer treatments; non-surgical cancer treatments can be neoadjuvant, adjuvant, or curative/mainline therapies	Studies that do not involve prehab or focus solely on rehabilitation during or after the completion of cancer treatments
	Outcomes of interest must be directly related to non-surgical cancer treatments and may include, but are not limited to, QoL, physical function, treatment adherence/tolerance, treatment-related side effects, or psychosocial well-being	Studies that focus exclusively on surgical prehab programs or only report on post-surgery outcomes
	Studies discussing the feasibility, acceptability, and implementation strategies of non-surgical cancer prehab programs	Studies that do not involve cancer patients or focus on non-cancer prehab programs
Context	Studies conducted in any clinical, community, or at-home settings	N/A
Other	English	Narrative reviews
	Articles published in any year	Systematic/scoping reviews
	Original research articles	Editorials
	Feasibility studies	Opinion/perspective papers
	Quantitative (RCTs, cohort study, case–control, cross-sectional studies, etc.), qualitative, and mixed-methods studies	Conference abstracts Study protocols/proposals

**Table 2:** Data extraction form.

Extraction Field	Details
General Information	Author(s), paper title, journal, year of publication, and source origin/country of origin
Population	Number of participants, participant age range, cancer type, and treatment type
Prehabilitation Intervention Used	Type of prehabilitation program (multimodal or unimodal), specific interventions used (e.g., exercise, nutrition therapy, and psychosocial support), duration of program, and program setting/context
Study Characteristics	Aim of prehab program/intervention, study design, study methodology, and outcomes measures
Outcome Results	Key findings that are related to the review question, including primary outcome results, qualitative analyses, barriers/enablers to program implementation, patient perspectives, and feasibility

**Table 3:** Baseline characteristics and intervention details of included studies.

Authors	Study Design	Disease Type	Cancer Treatment Type	N	Age	Prehab Type	Intervention Summary	Timing	Comparison
Aggarwal et al. [50]	Prospective single-arm cohort	Head and neck cancer	Curative RT or CTRT (radical or adjuvant)	30	Med 57	Unimodal (Exercise)	SLP-guided speech, voice, and swallowing exercises. Self-administered post-instruction.	Before (after complete staging), during, and post-treatment	None
Ajmani et al. [63]	Retrospective cohort	Head and neck cancer	Curative RT or CCRT (definitive or adjuvant)	254	Med 60	Unimodal (Exercise)	SLP swallowing program with targeted exercises.	Before, during, and 2–3 wks post-treatment	Historical group with reactive referral only
Bausys et al. [34]	RCT	Gastric cancer	Neoadjuvant chemo	122	Mean 61	Multimodal	Home-based exercise, respiratory and resistance training, nutrition, and relaxation.	1 wk before NAC through to surgery	Standard care without structured support
Blake et al. [64]	Retrospective pre-post cohort	Head and neck cancer	CTRT (mostly definitive)	111	Mean 60.7	Unimodal (Nutrition)	Dietetic counseling + enteral nutrition protocol via gastrostomy.	≥2 wks before CTRT through treatment until oral intake deemed adequate	Historical cohort without pre-treatment counseling
Brahmbhatt et al. [35]	Mixed methods feasibility RCT	Breast cancer	Neoadjuvant chemo	72	Mean 57.4	Multimodal	Unsupervised exercise, dietitian counseling, stress management, and smoking cessation.	Few wks before NAC through to surgery	Standard care
Büntzel et al. [71]	Non-randomized matched pair	Head and neck cancer	Curative RT	40	Med 58	Multimodal	Parenteral and oral nutrition + swallowing exercises.	2 wks pre-RT through treatment	Standard supportive care
Carmignani et al. [51]	Prospective case–control	Head and neck cancer	Curative or adjuvant RT/CTRT	12	Most 50–70	Unimodal (Exercise)	Home-based swallowing exercises, ten repetitions twice daily.	2 wks pre-treatment through 6 wks during treatment	Standard care without exercise protocol
Charters et al. [52]	Prospective single-arm cohort	Head and neck cancer (trismus)	Adjuvant RT	9	Mean 56.1	Unimodal (Exercise)	Jaw stretching using Restorabite device + SLP sessions.	3–6 wks post-surgery, pre-RT, continued 10 wks	None
Christodoulidis et al. [65]	Retrospective case–control	Esophageal/gastric junction cancer	Neoadjuvant chemo/CTRT	92	Mean 67.6	Multimodal	Home-based exercise + nutritional and psychological support.	Before NAC (at diagnosis) to surgery	Standard care per guidelines
Cnossen et al. [53]	Prospective single-arm feasibility	Head and neck cancer	Curative or adjuvant RT/CTRT	34	Mean 60	Multimodal	Pretreatment and educational counseling + home swallowing exercises with weekly coaching.	Up to 11 days pre-RT, 6 weeks during treatment	None
Di Franco et al. [54]	Prospective cohort	Breast cancer	Adjuvant RT ± chemo/hormone therapy	71	Mean 52.1	Unimodal (Nutrition)	6-week oral supplement (Ixor) + standard topical care.	10 days pre-RT to 10 days post-RT	Standard topical care only
Govender et al. [36]	Feasibility RCT	Head and neck cancer	RT/CTRT ± surgery	32	Mean 57	Multimodal	Targeted swallowing exercises + behavioral support + video education.	3–4 wks pre-RT through 6 months	General info + baseline assessment only
Halliday et al. [55]	Prospective single-arm cohort	Esophageal/gastric junction cancer	Neoadjuvant chemo	67	Mean 66	Unimodal (Exercise)	FITT-based home exercise program (aerobic + strength).	Before NAC (post-staging) until pre-surgery (16 wks)	None
Halliday et al. [66]	Retrospective cohort	Esophageal/gastric junction cancer	Neoadjuvant chemo/CTRT	79	Mean 66.2	Multimodal	Exercise + personalized nutrition with dietitian monitoring.	Start of NAC through pre-surgery (16 wks)	Standard care
Heiman et al. [37]	RCT	Breast cancer	Adjuvant therapies post-surgery (RT/chemo/endocrine therapy/bisphosphonate/anti-Her-2-therapy)	287	Med 63	Unimodal (Exercise)	Home aerobic exercise (30 min/day), self-paced + monitored.	2 wks pre-surgery (6–16 wks pre-adjuvant therapy) to 4 wks post-surgery	Standard physical activity advice only
Kotz et al. [38]	RCT	Head and neck cancer	Curative CTRT ± induction chemo/RT	26	Mean 59	Unimodal (Exercise)	Daily targeted swallowing exercises (five types).	Pre-CTRT through treatment	Standard care with reactive SLP referral
Liu et al. [39]	RCT	Liver cancer	RT	90	Mean 68.8	Multimodal	Nursing program with nutrition, Baduanjin exercise, meditation, and health education.	10–15 days pre-RT to start of treatment	Routine RT preparation
Loughney et al. [40]	RCT	Esophageal/gastric cancer	Neoadjuvant chemo/CTRT	71	Mean 62.2	Unimodal (Exercise)	Home or center-based aerobic and resistance training.	Pre-NAC through surgery; optional post-op phase	Standard care
Loughney et al. [41]	RCT	Esophageal/gastric cancer	Same as Loughney et al. [40]	71	Same as Loughney et al. [40]	Unimodal (Exercise)	Same program as Loughney et al. [40]; focus on behavior outcomes.	Same as Loughney et al. [40]	Same as Loughney et al. [40]
Malik et al. [67]	Retrospective cohort	Head and neck cancer	Curative or adjuvant RT/CTRT	1992	Mean 62.2	Multimodal	Group prehab class covering patient and family education, managing swallowing dysfunction, nutrition, and hydration.	Before or early RT	Non-attendees receiving standard care
Messing et al. [42]	RCT	Head and neck cancer	Curative CTRT	60	Med 56	Unimodal (Exercise)	Oromotor/swallow exercises + TheraBite protocol; weekly swallow-therapy sessions.	1 week pre-CTRT to 3 months post	No SLP contact; received prophylactic TheraBite only
Mortensen et al. [43]	RCT	Head and neck cancer	Curative RT/CTRT	44	Med 58	Unimodal (Exercise)	Daily prophylactic swallowing exercises + therapist visits.	Pre-RT to up to 9 months post-RT (11 months total)	Standard care with dietary advice only
Moug et al. [44]	Feasibility RCT	Rectal cancer	Neoadjuvant CTRT	48	Mean 65.9	Unimodal (Exercise)	Graduated walking program with biweekly step targets and 7 phone check-ins.	Before CTRT to 1–2 wks pre-surgery (13 wks total)	Standard care
Moug et al. [45]	Subanalysis of Moug et al. [44]	Rectal cancer	Neoadjuvant CTRT	44	Mean 66.8	Unimodal (Exercise)	Same as Moug et al. [44]	Same as Moug et al. [44]	Same as Moug et al. [44]
Natsume et al. [68]	Retrospective age-stratified cohort	Glioblastoma	Adjuvant CTRT post-surgery	75	Mean: older 72.5, younger 52.4	Multimodal	Physical rehab, aerobic and cognitive training, occupational therapy.	Post-op (up to 25 days pre-CTRT) to end of adjuvant CTRT (~6 weeks)	Two age groups (older and younger)
Ngo-Huang et al. [56]	Prospective single-arm cohort	Pancreatic cancer	Neoadjuvant chemo/CTRT	50	Mean 66.8	Multimodal	Moderate aerobic + strength training + dietitian counseling.	Pre-treatment to restaging before surgery	None
O’Loughlin et al. [57]	Prospective cohort	Pelvic (gynecologic) cancer	RT	28	Mean 63.4	Unimodal (Exercise)	Daily pelvic-focused exercises before each RT session.	Pre-RT (from CT simulation) through RT; optional post-treatment	Historical cohort without exercise
Phillips et al. [58]	Prospective cohort	Lung cancer	Radical systemic therapy (not otherwise specified)	243	Med 70	Multimodal	Symptom management + dietitian support + physio assessment/exercise.	Pre-diagnosis to post-treatment (no fixed end)	Historical cohort without intervention
Retel et al. [69]	Cost-effectiveness analysis	Head and neck cancer	Curative CTRT	90	Med 58	Unimodal (Exercise)	TheraBite mouth-opening + strengthening exercises (self-administered).	2 weeks pre-CTRT to 10 weeks post	Standard exercises without TheraBite
Retel et al. [70]	Cost-effectiveness analysis	Head and neck cancer	Curative CTRT	29	Med 57	Unimodal (Exercise)	Same TheraBite program as Retel et al. [69] but supervised.	2 weeks pre-CTRT to post-treatment	SLP-led standard exercises without TheraBite
Rossi et al. [72]	Program implementation report	Breast cancer	Neoadjuvant chemo ± other adjuvant therapies (chemo, RT, and endocrine therapy)	1500	NR	Multimodal	Lifestyle and diet counseling, psycho-oncology, physiotherapy, exercise complementary therapies (acupuncture, mindfulness, qigong, massage, and music/art therapy).	Pre-treatment (post-diagnosis) to surgery (~4–6 weeks)	None
Sacomori et al. [59]	Pilot prospective single cohort	Cervical cancer	Curative RT	49	Mean 44	Unimodal (Exercise)	Pelvic floor exercises; one supervised session + home-based program.	Up to 1 month pre-RT through treatment; up to 1 month post	None
Starmer et al. [60]	Prospective single cohort feasibility	Head and neck cancer	RT ± surgery/CTRT	36	Mean 61.9	Multimodal (exercise + tech)	SLP evaluation + swallowing exercises supported by Vibrent mobile app.	Up to 2 weeks pre-RT through 4-month period	None
Strijker et al. [61]	Prospective single cohort feasibility	Colorectal cancer	Adjuvant chemo post-surgery	16	Mean 62	Multimodal	Supervised endurance/resistance training + dietary counseling and supplementation.	Up to 24–38 days pre-chemo (within 2 wks post-surgery) through treatment	None
van der Molen et al. [46]	Feasibility RCT	Head and neck cancer	Curative CTRT	49	Mean 57	Unimodal (Exercise)	TheraBite mouth-opening protocol vs. standard swallowing exercises; same as Retel et al. [69]	Same as Retel et al. [69]	Same as Retel et al. [69]
van der Molen et al. [47]	RCT	Head and neck cancer	Curative CTRT	29	Med 60	Unimodal (Exercise)	TheraBite protocol vs. standard SLP program; same as Retel et al. [69]	Same as Retel et al. [69]	Same as Retel et al. [69]
Wang et al. [48]	RCT	Breast cancer	Adjuvant chemo post-surgery	72	Mean 50.4	Unimodal (Exercise)	6-week walking program based on self-efficacy model.	2–3 weeks pre-chemo (post-surgery) through mid-chemotherapy (6 wks total)	Usual care
Xu et al. [49]	Quasi-RCT	Breast cancer	Adjuvant chemo post-surgery	80	Mean 53.2	Multimodal (psychological)	8-week mindfulness-based cancer recovery program.	1 week before chemo through treatment	Routine nursing care
Zylstra et al. [62]	Prospective non-randomized controlled	Esophageal cancer	Neoadjuvant chemo pre-surgery	40	Med 64	Unimodal (exercise)	4-week walking + core/band/flexibility exercises guided by physio.	Pre-NAC through to surgery	Usual care

Note. RT: radiotherapy; CTRT: chemoradiotherapy; Med: median; SLP: speech–language pathology; RCT: randomized controlled trial; NAC: neoadjuvant chemotherapy; wks: weeks; FITT: Frequency, Intensity, Time, and Type of exercise; min: minutes; and NR: not reported. Age is reported for the full study cohort when available; otherwise, values reflect the intervention group. N is the number of participants in the entire study.

**Table 4:** Outcomes of included studies.

Authors	Outcome Time Points	Primary Outcomes	Key Findings
Aggarwal et al. [50]	Pre-RT, 4–6 wks post-RT	Speech, voice, and swallowing	Improved voice and speech after RT + prehab; swallowing did not improve until first follow-up.
Ajmani et al. [63]	Pre-RT, midpoint, and 2–3 wks post-treatment	SLP program feasibility, feeding tube placement	Increased pretreatment evaluations, reduced feeding tube placement, and improved oral intake.
Bausys et al. [34]	Pre-NAC, 1 wk pre-surgery, and 90 days post-surgery	Physical condition, fitness, nutrition status, QOL, and treatment adherence	Improved fitness, QOL, preoperative fitness, emotional functioning, and lower nonadherence to NAC in prehab group.
Blake et al. [64]	Pre-CTRT, 3 months post	Weight change, nutrition status, and feasibility	High feasibility (96% referral, 91% attendance, and 81% adherence); reduced weight loss and nutritional decline, but not statistically significant.
Brahmbhatt et al. [35]	Pre-NAC, 2 wks post-NAC, and 6 months post-surgery	Feasibility, 6MWT, QOL, and semi-structured interviews for participant experiences	Feasible with acceptable recruitment and low attrition; improved walking capacity, QOL, and less fatigue. Qualitative data supported high acceptability and emotional benefit.
Büntzel et al. [71]	Weekly during and end of RT	RT interruptions, duration, toxicities, and functional status	Fewer RT interruptions, lower toxicities, and better function in prehab group.
Carmignani et al. [51]	Baseline (2 wks pre-RT), 1 wk and 3 months post-RT	Swallowing, weight, and voice-related QOL	Improved swallowing, solid diet tolerance, and weight maintenance in prehab group.
Charters et al. [52]	Baseline, 10 and 26 wks post-intervention	Feasibility, trismus-related function, mouth opening, and QOL	Safe (no adverse events) and feasible (100% retention); significant improvement in mouth opening and trismus-related QOL.
Christodoulidis et al. [65]	Post-NAC	Chemo completion rate	Prehabilitation improved chemotherapy completion rate vs. controls (93.6% vs. 77.7%).
Cnossen et al. [53]	During and post-intervention	Feasibility, adherence, and barriers/facilitators	Feasible with good uptake, reasonable adherence, and moderate-to-high level of exercise performance. Common barriers included poor physical condition, treatment side effects, fatigue, and low motivation; facilitators included improved physical condition, motivation, and social or technical support.
Di Franco et al. [54]	Weekly during RT	Skin toxicity (Grade 2–3)	Ixor reduced skin toxicity especially in patients with moderate radiation doses (OR 0.50), breast volume < 500 mL, and those undergoing chemo with anthracyclines or taxanes (OR 0.68).
Govender et al. [36]	Baseline (pre-tx), 1, 3, and 6 months post-intervention start	Feasibility, adherence, and swallowing QOL	Feasible with 91% retention and good adherence; MDADI identified as key outcome.
Halliday et al. [55]	Baseline (pre-NAC), post-NAC, and 1 wk pre-surgery	Cardiorespiratory fitness, physical activity, and adherence	Preserved fitness during NAC and improved fitness pre-surgery; higher adherence linked to greater gains.
Halliday et al. [66]	Pre- and post-NAC	Body composition, hand grip strength, and exercise volume	Less skeletal muscle loss in prehab group; greater activity linked to reduced visceral fat. No change in hand grip strength between diagnosis and post-NAC.
Heiman et al. [37]	Baseline (pre-tx), 4 weeks and 12 months post-surgery	Quality of life	No QOL differences by group, but adjuvant chemo patients had lower QOL at 12 months (OR 0.475).
Kotz et al. [38]	Baseline, 1 week to 12 months post-CTRT	Swallowing function (PSS-H&N, FOIS)	Better swallowing function at 3–6 months post-CTRT in prehab group; no difference immediately after CRTR or post 9–12 months.
Liu et al. [39]	Baseline (10–15 days pre-RT) and 1 day post-RT	Grip strength, albumin, QOL, and immune markers	Improved QOL, immune function, and nutrition status in prehab group.
Loughney et al. [40]	Pre-NAC, post-NAC, and pre-surgery	6MWT, body composition, and strength	Improved 6MWT in prehab group; no difference in secondary outcomes.
Loughney et al. [41]	Same as Loughney et al. [40]	Physical activity and sedentary behavior	No significant changes in activity or sedentary time between groups.
Malik et al. [67]	2-year follow-up post-tx	Overall/locoregional recurrence-free survival, toxicities, and treatment completion	Higher overall and locoregional recurrence-free survival, lower treatment complications in prehab group; no toxicity difference.
Messing et al. [42]	Baseline (pre-tx), 3, 6, 12, and 24 months post-CTRT	Functional oral intake, oromotor function, and swallow efficiency	Improved early oromotor function, pharyngeal impairment, swallow efficiency, and incisal opening at 3–6 months; functional oral intake gains not statistically significant.
Mortensen et al. [43]	Baseline (pre-tx), 2, 5, and 11 months post-RT	Swallow score, QOL, and mouth opening	No significant difference in swallowing outcomes between groups; high dropout and moderate adherence.
Moug et al. [44]	Baseline (pre-tx) to 1–2 wks pre-surgery	Feasibility, adherence, and step counts	Feasible, with good adherence, patient satisfaction, and no serious adverse events; smaller decline in step count vs. control, but not significant.
Moug et al. [45]	Same as Moug et al. [44]	Muscle mass (psoas area)	More patients in the prehab group gained muscle mass; trend toward preserving lean mass.
Natsume et al. [68]	Post-op, end of CTRT, and follow-up	Survival, functional status, and ADL	Improved functional outcomes and ADLs regardless of age; while there were more adverse events in the older group, there was no survival difference by age group.
Ngo-Huang et al. [56]	Baseline (pre-intervention) to restaging pre-surgery	Objective physical function (6MWT, handgrip strength, and 5xSTS), QOL, and determination of frailty	Significant gains in function and health-related QOL; sedentary behavior linked to worse QOL. No significant difference in objective outcomes by sarcopenia status or frailty status.
O’Loughlin et al. [57]	Baseline (simulation CT), each RT week, and 6–20 months post-RT	Sacral slope angle (SSA) variability, setup reproducibility	Improved positioning stability in prehab group (lower SSA variability).
Phillips et al. [58]	6 weeks post-diagnosis, 1- and 2-year survival	ER visits, hospital stays, and treatment rates	Fewer admissions, shorter hospital stays, and higher short-term survival in prehab group.
Retel et al. [69]	12 months post-CTRT	Cost, QALY, tube dependency, and number of hospital admissions	Lower tube dependency, fewer hospital admission days, and better QALYs despite higher cost; 83% cost-effective.
Retel et al. [70]	2 years post-CTRT	Cost, QALY, trismus, functional oral intake, and facial pain	Lower costs, better outcomes in TheraBite group (lower trismus, aspiration, and diet restriction rates), and more QALY; 70% probability cost-effective.
Rossi et al. [72]	Program activity metrics only	Uptake, activity volume	High feasibility and uptake; of 1500 patients, 83% underwent lifestyle counseling and 85% a psycho-oncological consultation before NAC. Overall, 1780 acupuncture treatments, 1340 physiotherapy sessions, and 218 herbal medicine counseling sessions have been carried out. A total of 90 patients completed the mindfulness-based stress reduction protocol and 970 participated in qi gong, art therapy, or music therapy classes.
Sacomori et al. [59]	Baseline (1 month pre-RT) and 1 month post-RT	Pelvic floor strength, activation of pelvic floor muscle, urinary incontinence, and feasibility/adherence	No significant improvements in pelvic floor muscle strength, activation, and incontinence, but it was feasible and may protect muscle function during RT.
Starmer et al. [60]	3-month app use period	Feasibility, adherence, satisfaction, and usability	App use was feasible; 80% used it, though adherence declined over time.
Strijker et al. [61]	Baseline (pre-chemo) through end of adjuvant chemo	Feasibility, adherence, safety, and participant satisfaction	Program was feasible and well accepted by participants, with 84% enrolment and high adherence (97% phase 1, 83% phase 2); no adverse events.
van der Molen et al. [46]	Baseline (1–2 wks pre-CTRT) and 10 wks post-CTRT	Feasibility, compliance, and functional outcomes	Moderate adherence and functional decline in both groups; fewer tube-dependent patients overall.
van der Molen et al. [47]	Baseline (1–2 wks pre-CTRT), 10 weeks, 1 and 2 years post-CTRT	Videofluoroscopy, functional outcomes	Most tumor- and treatment-related impairments improved by year 1; at 2 years, the only additional improvement was weight in TheraBite group.
Wang et al. [48]	Baseline (24 hrs pre-surgery), 24 hrs pre-chemo (2–3 wks post-surgery), and mid-cycle chemo (7–10 days after chemo initiation), to end of 6-week program	QOL, fatigue, sleep, self-efficacy, and activity	Significant improvements in QOL, fatigue, sleep disturbances, exercise self-efficacy, exercise behavior, and exercise capacity vs. control by mid-chemo (*p* < 0.001); program was effective and feasible.
Xu et al. [49]	Pre- and post-intervention	Anxiety, depression, PTSD, and fatigue	Mindfulness program reduced psychological distress and fatigue, with stronger effects in prehab group (all *p* < 0.05).
Zylstra et al. [62]	Baseline (pre-NAC), 1–7 days post-NAC, and 1–7 days pre-surgery	Tumor regression, body composition, and immune markers	Higher tumor regression, greater combined tumor and nodal downstaging, less fat loss, and better immune markers in prehab group.

Note. RT: radiotherapy; SLP: speech–language pathology; NAC: neoadjuvant chemotherapy; QOL: quality of life; CTRT: chemoradiotherapy; 6MWT: 6 min walk test; wks: weeks; PSS-H&N: performance status scale for head and neck cancer patients; FOIS: functional oral intake scale; tx: treatment; ADLs: activities of daily living; 5xSTS: 5 times stand sit-to-stand test; ER: emergency room; QALY: quality-adjusted life years; and PTSD: post-traumatic stress disorder.

## Data Availability

No new data were created or analyzed in this study.

## Supplementary Materials

The following supporting information can be downloaded at https://www.mdpi.com/article/10.3390/cancers18020286/s1, Table S1: Ovid 1946 to 22 October 2024.
