# Supplementary material for: Prehabilitation in Adult Cancer Patients Undergoing Chemotherapy or Radiotherapy: A Scoping Review
Source: Cancers (Basel). 2026 Jan 16;18(2):286. doi: 10.3390/cancers18020286 (PMC12838628; doi:10.3390/cancers18020286)
Supplement: Supplementary file 1 [file cancers-18-00286-s001.zip › cancers-4068024-supplementary.pdf]

cancers

MDPI

Supplementary Materials: Prehabilitation in Adult Cancer Patients Undergoing Chemotherapy or Radiotherapy: A Scoping Review

Dylan Kwan, Wesley Kwan, Anchal Badwal, Tuti Puol, Justin Zou Deng, Raymond Wang, Saad Ahmed, Alexandria Mansfield, Rouhi Fazelzad and Jennifer Jones

Table S1. Ovid 1946 to 22 October 2024.

#	Searches	Results	Type
1	exp Neoplasms/	4032937	Advanced
2	exp Radiotherapy/	214200	Advanced
3	exp Antineoplastic Agents/	1294009	Advanced
4	exp Antineoplastic Protocols/	166885	Advanced
5	Antineoplastic Combined Chemotherapy Protocols/	166334	Advanced
6	exp Chemotherapy, Adjuvant/	47965	Advanced
7	Consolidation Chemotherapy/	791	Advanced
8	Induction Chemotherapy/	4105	Advanced
9	Maintenance Chemotherapy/	2246	Advanced
10	Maintenance Chemotherapy/	2246	Advanced
11	neoplas*.tw,kf.	472542	Advanced
12	paraneoplas*.tw,kf.	13780	Advanced
13	cancer*.tw,kf.	2460564	Advanced
14	tumo?r*.tw,kf.	2182796	Advanced
15	onco*.tw,kf.	453444	Advanced
16	metast*.tw,kf.	676092	Advanced
17	multimetast*.tw,kf.	54	Advanced
18	macrometast*.tw,kf.	1164	Advanced
19	micrometast*.tw,kf.	7470	Advanced
20	malig*.tw,kf.	741516	Advanced
21	aberrant crypt foci.tw,kf.	1627	Advanced
22	acanthoma*.tw,kf.	776	Advanced
23	acrospiroma*.tw,kf.	91	Advanced
24	adamantinom*.tw,kf.	1332	Advanced
25	adenocarc*.tw,kf.	195631	Advanced
26	adenofibrom*.tw,kf.	607	Advanced
27	adenolymphom*.tw,kf.	481	Advanced
28	adenomat*.tw,kf.	21128	Advanced
29	adenomyo*.tw,kf.	5561	Advanced
30	adenosarcom*.tw,kf.	696	Advanced
31	adenosquam*.tw,kf.	3402	Advanced
32	ameloblastom*.tw,kf.	4184	Advanced
33	androblastom*.tw,kf.	99	Advanced
34	angiofibrom*.tw,kf.	2548	Advanced
35	angiokeratom*.tw,kf.	1141	Advanced
36	angiolipom*.tw,kf.	720	Advanced
37	angioma*.tw,kf.	12277	Advanced
38	angiomyolipom*.tw,kf.	4804	Advanced
39	angiomyom*.tw,kf.	178	Advanced
40	angiosarcom*.tw,kf.	7892	Advanced
41	apudoma*.tw,kf.	302	Advanced
42	arrhenoblastom*.tw,kf.	355	Advanced
43	astrocytom*.tw,kf.	18742	Advanced

 https://doi.org/10.3390/cancers18020286

44	blastom*.tw,kf.	10604	Advanced
45	Bowen*.tw,kf.	3587	Advanced
46	Brenner*.tw,kf.	1695	Advanced
47	Buschke-Lowenstein*.tw,kf.	316	Advanced
48	carcin*.tw,kf.	993223	Advanced
49	cementoma*.tw,kf.	225	Advanced
50	chemodectomas*.tw,kf.	314	Advanced
51	cholangiocarcin*.tw,kf.	20354	Advanced
52	chondroblastom*.tw,kf.	1225	Advanced
53	chondroma*.tw,kf.	4811	Advanced
54	chordoma*.tw,kf.	5035	Advanced
55	chondrosarcom*.tw,kf.	9596	Advanced
56	choriocarcin*.tw,kf.	7728	Advanced
57	craniopharyngioma*.tw,kf.	5393	Advanced
58	cystadenofibrom*.tw,kf.	240	Advanced
59	cystosarcom*.tw,kf.	651	Advanced
60	cytoma*.tw,kf.	403	Advanced
61	dermatofibrosarcom*.tw,kf.	2443	Advanced
62	desmoplas*.tw,kf.	6184	Advanced
63	dysgerminoma*.tw,kf.	1544	Advanced
64	DCIS.tw,kf.	6257	Advanced
65	DSRCT.tw,kf.	447	Advanced
66	ependymom*.tw,kf.	6469	Advanced
67	Ewing*.tw,kf.	12029	Advanced
68	fibroadenom*.tw,kf.	4419	Advanced
69	fibroepithelial*.tw,kf.	1381	Advanced
70	fibroma*.tw,kf.	14899	Advanced
71	fibrosarcom*.tw,kf.	12798	Advanced
72	FAMMM.tw,kf.	88	Advanced
73	gangliogliom*.tw,kf.	1670	Advanced
74	ganglioneurom*.tw,kf.	2517	Advanced
75	gastrinoma*.tw,kf.	1840	Advanced
76	germinoma*.tw,kf.	2227	Advanced
77	glioblastom*.tw,kf.	55167	Advanced
78	glioma*.tw,kf.	77488	Advanced
79	gliosarcom*.tw,kf.	1276	Advanced
80	glomus jugulare*.tw,kf.	908	Advanced
81	glomus tympanicum*.tw,kf.	240	Advanced
82	glucagonoma*.tw,kf.	1016	Advanced
83	gonadoblastom*.tw,kf.	912	Advanced
84	GCTOB.tw,kf.	11	Advanced
85	GIST?.tw,kf.	10377	Advanced
86	h?emangioendotheliom*.tw,kf.	4061	Advanced
87	h?emangiom*.tw,kf.	27331	Advanced
88	h?emangiopericytom*.tw,kf.	3743	Advanced
89	h?emangiosarcom*.tw,kf.	1547	Advanced
90	hamartoblastom*.tw,kf.	49	Advanced
91	hepatoblastom*.tw,kf.	4375	Advanced
92	hepatoma*.tw,kf.	31005	Advanced
93	histiocytom*.tw,kf.	6028	Advanced
94	hodgkin*.tw,kf.	76664	Advanced
95	nonhodgkin*.tw,kf.	139	Advanced
96	(hutchinson* adj2 freckle*).tw,kf.	63	Advanced
97	HNPCC.tw,kf.	2339	Advanced
98	immunocytom*.tw,kf.	619	Advanced
99	incidentaloma?.tw,kf.	3015	Advanced
100	insulinoma*.tw,kf.	7424	Advanced
101	kasabach merrit*.tw,kf.	882	Advanced
102	leiomyoblastom*.tw,kf.	404	Advanced

https://doi.org/10.3390/cancers18020286

103	leiomyom*.tw,kf.	18007	Advanced
104	leiomyosarcom*.tw,kf.	12140	Advanced
105	leuk?em*.tw,kf.	317359	Advanced
106	preleuk?em*.tw,kf.	1856	Advanced
107	leukoplak*.tw,kf.	5720	Advanced
108	li-fraumeni*.tw,kf.	1694	Advanced
109	lipoblastom*.tw,kf.	595	Advanced
110	lipoma*.tw,kf.	17433	Advanced
111	liposarcom*.tw,kf.	8273	Advanced
112	luteoma*.tw,kf.	257	Advanced
113	lymphangio*.tw,kf.	15968	Advanced
114	lymphoblastom*.tw,kf.	375	Advanced
115	lymphocytom*.tw,kf.	374	Advanced
116	lymphoma*.tw,kf.	219283	Advanced
117	lymphosarcom*.tw,kf.	5250	Advanced
118	lynch*.tw,kf.	6162	Advanced
119	macroglobulinem*.tw,kf.	4431	Advanced
120	m?croprolactinom*.tw,kf.	976	Advanced
121	mastocytom*.tw,kf.	1925	Advanced
122	mastocytos?s*.tw,kf.	4607	Advanced
123	medulloblastom*.tw,kf.	10202	Advanced
124	meigs*.tw,kf.	967	Advanced
125	melanoameloblastom*.tw,kf.	10	Advanced
126	melanoblastom*.tw,kf.	476	Advanced
127	melanocarcin*.tw,kf.	104	Advanced
128	melanoma*.tw,kf.	148461	Advanced
129	melanosis.tw,kf.	2815	Advanced
130	melanotic*.tw,kf.	2853	Advanced
131	meningiom*.tw,kf.	26310	Advanced
132	mesenchymom*.tw,kf.	871	Advanced
133	mesoblast*.tw,kf.	809	Advanced
134	mesonephrom*.tw,kf.	123	Advanced
135	mesotheliom*.tw,kf.	19584	Advanced
136	metaplas*.tw,kf.	26535	Advanced
137	muir-torre*.tw,kf.	564	Advanced
138	myelolipom*.tw,kf.	1283	Advanced
139	myoepitheliom*.tw,kf.	872	Advanced
140	myofibrom*.tw,kf.	811	Advanced
141	myeloma*.tw,kf.	67569	Advanced
142	myoma*.tw,kf.	7155	Advanced
143	myosarcom*.tw,kf.	239	Advanced
144	myxofibrosarcom*.tw,kf.	832	Advanced
145	myxoma*.tw,kf.	10752	Advanced
146	myxosarcom*.tw,kf.	286	Advanced
147	n?evocarcin*.tw,kf.	78	Advanced
148	neurilemmom*.tw,kf.	2451	Advanced
149	neurocytom*.tw,kf.	891	Advanced
150	neuroectodermal*.tw,kf.	7502	Advanced
151	neurofibroma*.tw,kf.	21545	Advanced
152	neurofibrosarcom*.tw,kf.	431	Advanced
153	neurilemmom*.tw,kf.	2451	Advanced
154	neuroblastom*.tw,kf.	42996	Advanced
155	neuroma*.tw,kf.	11734	Advanced
156	neurothekeom*.tw,kf.	287	Advanced
157	NSCLC.tw,kf.	67578	Advanced
158	odontoma*.tw,kf.	1690	Advanced
159	oligo*.tw,kf.	315208	Advanced
160	osteoblastom*.tw,kf.	1343	Advanced
161	osteochondrom*.tw,kf.	3733	Advanced

https://doi.org/10.3390/cancers18020286

162	osteoclastom*.tw,kf.	395	Advanced
163	osteoma*.tw,kf.	12594	Advanced
164	osteosarcom*.tw,kf.	31126	Advanced
165	papilloma*.tw,kf.	74321	Advanced
166	papillary*.tw,kf.	69891	Advanced
167	paragangliom*.tw,kf.	9486	Advanced
168	pheochromocytom*.tw,kf.	20259	Advanced
169	phyllo?des*.tw,kf.	2805	Advanced
170	pinealocytoma*.tw,kf.	18	Advanced
171	pinealoma*.tw,kf.	366	Advanced
172	pineoblastoma*.tw,kf.	530	Advanced
173	pineocytoma*.tw,kf.	295	Advanced
174	plasmacytom*.tw,kf.	7542	Advanced
175	(polycythem* adj2 vera?).tw,kf.	6699	Advanced
176	prolactinom*.tw,kf.	4016	Advanced
177	retinoblastom*.tw,kf.	18738	Advanced
178	rhabdoid*.tw,kf.	3610	Advanced
179	rhabdomyom*.tw,kf.	1814	Advanced
180	rhabdomyosarcom*.tw,kf.	14129	Advanced
181	sarcom*.tw,kf.	131804	Advanced
182	seminoma*.tw,kf.	8293	Advanced
183	Sertoli- Leydig.tw,kf.	795	Advanced
184	somatostatinoma*.tw,kf.	407	Advanced
185	somatotrophinom*.tw,kf.	89	Advanced
186	struma ovarii*.tw,kf.	709	Advanced
187	thecoma*.tw,kf.	499	Advanced
188	teratocarcin*.tw,kf.	2795	Advanced
189	teratoma*.tw,kf.	17733	Advanced
190	thymom*.tw,kf.	11243	Advanced
191	trophoblast*.tw,kf.	27271	Advanced
192	vipoma*.tw,kf.	432	Advanced
193	wilms*.tw,kf.	11484	Advanced
194	(radiotherap* or radio-therap*).tw,kf.	238431	Advanced
195	(chemotherap* or chemo-therap*).tw,kf.	537806	Advanced
196	(chemo or CTx).tw,kf.	47379	Advanced
197	(chemorad* or chemo-rad*).tw,kf.	44896	Advanced
198	or/1-197	6706524	Advanced
199	Rehabilitation/	18786	Advanced
200	Telerehabilitation/	1285	Advanced
201	(prehab* or pre-hab*).tw,kf.	2213	Advanced
202	(prerehab* or pre-rehab*).tw,kf.	206	Advanced
203	(early and (rehab* or habilit*)).tw,kf.	25516	Advanced
204	((multimodal* or multi-modal*) and (rehab* or habilit*)).tw,kf.	2592	Advanced
205	((preinterven* or pre-interven*) and (rehab* or habilit*)).tw,kf.	515	Advanced
206	((before adj3 interven*) and (rehab* or habilit*)).tw,kf.	942	Advanced
207	((prior adj3 interven*) and (rehab* or habilit*)).tw,kf.	192	Advanced
208	((pretreat* or pre-treat*) and (rehab* or habilit*)).tw,kf.	996	Advanced
209	((before adj3 treat*) and (rehab* or habilit*)).tw,kf.	2068	Advanced
210	((prior adj3 treat*) and (rehab* or habilit*)).tw,kf.	357	Advanced
211	((pretherap* or pre-therap*) and (rehab* or habilit*)).tw,kf.	48	Advanced
212	((before adj3 therap*) and (rehab* or habilit*)).tw,kf.	419	Advanced
213	((prior adj3 therap*) and (rehab* or habilit*)).tw,kf.	120	Advanced
214	((preempt* or pre-empt*) and (rehab* or habilit*)).tw,kf.	156	Advanced
215	(prevent* and (rehab* or habilit*)).tw,kf.	21235	Advanced
216	(proact* and (rehab* or habilit*)).tw,kf.	548	Advanced
217	(prophyl* and (rehab* or habilit*)).tw,kf.	1275	Advanced
218	(precaution* and (rehab* or habilit*)).tw,kf.	407	Advanced
219	((distan* or remote*) adj3 (rehab* or re-hab*)).tw,kw.	545	Advanced
220	(telerehab* or tele-rehab*).tw,kf.	2925	Advanced

https://doi.org/10.3390/cancers18020286

221	(erehab* or mrehab* or trehab*).tw,kf.	29	Advanced
222	(teleprehab* or telepre-hab*).tw,kf.	14	Advanced
223	(eprehab* or mprehab* or tprehab*).tw,kf.	0	Advanced
224	or/199-223	73647	Advanced
225	198 and 224	5659	Advanced
226	((prechemo* or pre-chemo*) and (rehab* or habilit*)).tw,kf.	5	Advanced
227	((before adj3 chemo*) and (rehab* or habilit*)).tw,kf.	35	Advanced
228	((prior adj3 chemo*) and (rehab* or habilit*)).tw,kf.	9	Advanced
229	((prerad* or pre-rad*) and (rehab* or habilit*)).tw,kf.	13	Advanced
230	((before adj3 rad*) and (rehab* or habilit*)).tw,kf.	130	Advanced
231	((prior adj3 rad*) and (rehab* or habilit*)).tw,kf.	57	Advanced
232	or/225-231	5809	Advanced
233	limit 232 to "humans only (removes records about animals)"	5750	Advanced
234	limit 233 to "all child (0 to 18 years)"	563	Advanced
235	limit 233 to "all adult (19 plus years)"	2380	Advanced
236	234 not 235	245	Advanced
237	233 not 236	5505	Advanced
238	limit 237 to (clinical conference or clinical trial, veterinary or clinical trial protocol or consensus development conference or consensus development conference, nih or meta analysis or news or newspaper article or retracted publication or "retraction of publication" or "review" or "scientific integrity review" or "systematic review")	1377	Advanced
239	237 not 238	4128	Advanced
240	limit 239 to english language	3512	Advanced

Disclaimer/Publisher's Note: The statements, opinions and data contained in all publications are solely those of the individual author(s) and contributor(s) and not of MDPI and/or the editor(s). MDPI and/or the editor(s) disclaim responsibility for any injury to people or property resulting from any ideas, methods, instructions or products referred to in the content.

https://doi.org/10.3390/cancers18020286
